# Mitral valve replacement for rheumatic heart disease in Southern Africa

**DOI:** 10.1186/1749-8090-8-S1-O294

**Published:** 2013-09-11

**Authors:** P Zilla, J Koshy, J Brink, P Human

**Affiliations:** 1 University of Cape Town, Cape Town, South Africa

## Background

Threshold countries like South Africa provide cardiac surgery to a largely indigent population with rheumatic heart disease. Although repairs are a preferred treatment modality many rheumatic mitral valves can only be replaced. In view of significantly improved primary health care and broad access of the indigent population to communication technology we revisited the efficacy of mitral valve replacement (MVR) at the interface of the developing and developed world.

## Methods

A cohort of 280 patients (mean age 40.7±13.7y/range 12-80y/median 41y; 76.4% female) with rheumatic heart disease (21% MR; 11% MS; 68% mixed) undergoing mitral valve replacement (MVR) (88.2% mechanical versus 11.8% tissue valves) was analyzed.

## Results

Follow-up for the entire cohort was 94% complete (median follow-up period 3.5 years). Actuarial 5-year freedom from valve related reoperation/death was 81.5±2.9%/96.7±1.3% in the mechanical and 81.8±6.7% /100±0% in the tissue valve group (p=0.562/0.970). There was no significant difference in freedom from death or reoperation irrespective of whether INR tests were performed or not. In the mechanical group, partition modeling demonstrated a significant difference with respect to freedom from death (95.3±3.2% versus 82.9±4.1% %; p=0.013) but not with respect to freedom from reoperation (98.2±1.8% versus 96.2±3.8%; p=0.329) between patients divided by an INR cut point of 34% of tests falling within the therapeutic range. Compared to the tissue valve group, the mechanical group with poor INR control was statistically equivalent with respect to survival and reoperation rate.

## Conclusion

Overall circumstances for patients with rheumatic heart disease needing mitral valve replacement in a developing country have become conducive for the use of mechanical prostheses. Yet, the remaining inferior outcome in patients with suboptimal INR control highlights the need for alternative anticoagulation agents that require less stringent dosing.

